# Integration of real-world evidence from different data sources in health technology assessment

**DOI:** 10.3389/jpps.2023.11460

**Published:** 2023-07-17

**Authors:** Pooyeh Graili, Jason R. Guertin, Kelvin K. W. Chan, Mina Tadrous

**Affiliations:** ^1^ Leslie Dan Faculty of Pharmacy, University of Toronto, Toronto, ON, Canada; ^2^ Quality HTA, Oakville, ON, Canada; ^3^ Axe Santé des Populations et Pratiques Optimales en Santé, Centre de Recherche du CHU de Québec-Université Laval, Laval, QC, Canada; ^4^ Department of Social and Preventive Medicine, Université Laval, Laval, QC, Canada; ^5^ Tissue Engineering Laboratory (LOEX), Université Laval, Laval, QC, Canada; ^6^ Sunnybrook Health Science Centre, Toronto, ON, Canada; ^7^ Department of Medicine, University of Toronto, Toronto, ON, Canada; ^8^ Canadian Centre for Applied Research in Cancer Control, Toronto, ON, Canada; ^9^ Women’s College Research Institute, Women’s College Hospital, Toronto, ON, Canada

**Keywords:** real-world evidence, health technology, health technology assessment, real-world data, reassessment

## Abstract

Real-world evidence (RWE) is being increasingly used by a wide range of stakeholders involved in the therapeutic product lifecycle but remains underutilized in the health technology assessment (HTA) process. RWE aims to fill the current evidence gaps, reduce the uncertainty around the benefits of medical technologies, and better understand the long-term impact of health technologies in real-world conditions. Despite the minimal use of RWE in some elements of HTA, there has been a larger push to further utilize RWE in the HTA processes. HTA bodies, as other stakeholders, work towards developing more robust means to leverage RWE from various data sources in the HTA processes. However, these agencies need to overcome important challenges before the broader incorporation of RWE into their routine practice. This paper aims to explore the extensive integration of RWE utilizing diverse sources of RWD. We discuss the utilization of RWE in HTA processes, considering aspects such as when, where, and how RWE can be effectively applied. Additionally, we seek the potential challenges and barriers associated with the utilization of different data sources.

## Introduction

The potential value of Real-World Evidence (RWE) has garnered a great deal of global attention from a range of stakeholders, such as decision-makers, researchers, practitioners, patients, and manufacturers [1–4]. In the decision-making sphere for health technologies, regulatory agencies such as the Food and Drug Administration and European Medicines Agency have historically used RWE to evaluate post-marketing safety and are now working towards expanding its use to support the approval process in limited circumstances [5]. Many regulators have developed frameworks over the last few years to enable and optimize the use of RWE in regulating health technologies [6–8].

Despite recent initiatives to use RWE in regulatory processes, health technology assessment (HTA) agencies have not yet fully integrated RWE into their routine processes. HTA agencies, like National Institute for Health Care Excellence (NICE) in England, the Canadian Agency for Drug and Technology in Health (CADTH), Haute Autorité de santé (HAS) in France, and Institut für Qualität und Wirtschaftlichkeit im Gesundheitswesen/Der Gemeinsame Bundesausschuss (IQWIG/G-BA) in Germany, are moving toward optimal use of RWE in their HTA processes. France, also uses RWE to reassess the technologies reimbursed based on randomized controlled trials (RCTs). Other HTA and reimbursement agencies in Asian countries including Japan, Taiwan, and South Korea rely on RWE to adjust prices and reassess funded technologies. Some other countries like India, China, and Singapore make reimbursement decisions based on RWE several years after market entry [9, 10]. The current RWE in the HTA realm has largely been limited to observational data and registries to better understand the epidemiology, natural course and burden of disease, safety, effectiveness, and long-term impact of technologies, limited inputs for pharmacoeconomics and budget impact analysis models, surveys and interviews for patients’ experiences [10–12]. Still, standard frameworks to use a wide range of RWE from different emerging sources in routine HTA practice globally are lacking. Recently, some HTA bodies, such as HAS, NICE, CADTH and the Institut national d’excellence en santé et en services sociaux (INESSS) have initiated developing RWE frameworks to broaden the use of RWE in HTA. HAS developed guidelines on using RWE in HTA in 2021, while NICE released its RWE framework as a guide for good practice in 2022 [11]. CADTH has also developed a national framework to optimize the use of RWE collaboratively with INESSS and the Canadian regulatory body, Health Canada, in 2022 [13]. As HTA bodies work towards developing more robust means to leverage RWE from various data sources, there are important challenges and opportunities to consider [4, 6, 7, 13, 14]. The objective of this paper is to explore the integration of RWE utilizing diverse sources of real-world data (RWD) in HTA processes. This paper discusses the utilization of RWE in HTA processes, considering aspects such as when, where, and how RWE can be effectively applied. Furthermore, it aims to discuss the potential challenges and barriers that arise associated with the utilization of different data sources.

## What is RWE?

RWE is derived from RWD and characterized by the routine use of health technologies in real life outside of the clinical trial setting [15]. RWD are collected from different sources [2, 3, 5, 11, 16] and can be quantitative or qualitative. They contain information pertaining to a patient’s medical history, demographics, clinical outcomes, lab data, imaging information, resource use and costs, health behaviours and/or patient experiences [2, 4, 11].

**FIGURE 1:**
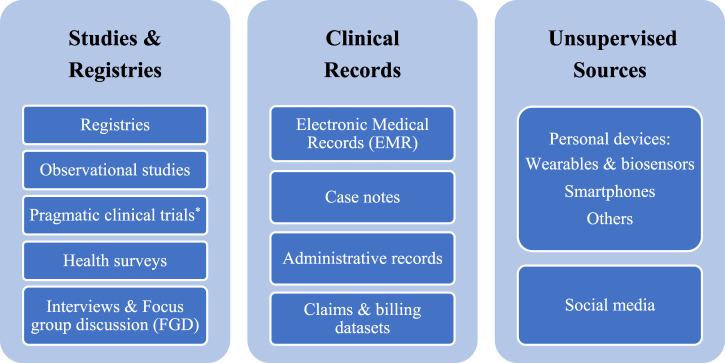
Real-world data sources. * These trials, unlike traditional clinical trials, provide the evidence to address the impact of health interventions on real-world practice without the inclusion and exclusion criteria defined in the protocols of clinical trials.

Each RWD source supports certain evidence gaps and could help answer a wide swath of research questions on different elements of the HTA requirements. The diversity of RWD sources is an exciting opportunity, and data sources can be categorized into three main groups based on the level of their potential quality: data originating from “studies and registries,” “clinical records,” and “unsupervised sources” (Figure 1). The “studies and registries” category includes sources where data are collected purposefully for analysis using scientific methods and defined protocols. Collected data from these sources of the highest quality that positively impacts data integrity and analysis. The “clinical records” category consists of sources with data originating from routine medical care without following study protocols but under the supervision of healthcare professionals. The “unsupervised sources” category contains a wide range of sources containing data collected without the supervision of any trained healthcare professional or not as part of any protocol. There are established protocols and methodological solutions to tackle uncertainty around the challenges of using RWD sources from “studies and registries.” While developing standard frameworks as well as robust and responsive methodologies to overcome the challenges of data from “clinical records” and “unsupervised sources” are required. Given the diversity of RWD sources, it is essential to understand RWD quality and have full transparency on how data is collected, cleaned, curated, and linked before using RWE in the HTA processes.

RWE is broadly defined as any evidence obtained from RWD. Traditionally, RWE has been called observational research, as it uses observational data to investigate the effect of an intervention retrospectively or prospectively. These evidence can be represented in a variety of study designs [17]. Depending on the research question, their analysis methods may vary from simple regression to complex multivariable regression and time-series analyses. Given the real-world nature of these studies, there are often concerns with operational, technical, and methodological challenges which should be resolved in advance. The operational concerns include feasibility, governance, and sustainability, which can be addressed with data sharing agreement during the initiation of the study inception and data anonymization processes as needed. The methodological issues arise from confounders, different biases, and amounts of missing data. The solutions require focusing on a detailed description of study design, registration of the study, and employing established statistics and epidemiology techniques to leverage bias reduction and control for potential confounders. This is especially important in questions aiming to assess correlation, such as those pertaining to real-world effectiveness and safety. The other issues, technical challenges, are mainly related to data. Some potential solutions can be quality assurance and following common data formats and terminologies. On top of all these challenges, HTA bodies may face other hurdles when it comes to leveraging this type of RWE. Among them is the issue of data insufficiency, where the selected sample of patients may not accurately represent those found in daily clinical practice. Furthermore, HTA procuresses can be hindered by challenges such as incomplete reporting and missing other treatments and relevant outcomes, and other insufficient evidence in the HTA dossiers [9, 18]. Thus, this type of traditional RWE can cause uncertainty in the decision-making processes due to their potential limitations. We need various sources of RWE to complement trial findings and provide additional information related to practice patterns and patient characteristics in real-world settings [5].

## Why does RWE matter?

Currently, it is standard practice in HTA that many sponsors submit their dossiers using clinical trials and potentially include indirect comparison evidence as a demonstration of the benefits of their products to HTA bodies. RCTs are still the gold standard of evidence and are preferred to assess innovative technologies’ safety, efficacy, and effectiveness before launch. Apparently, despite low external validity because of the lack of generalizability, RCTs have a high level of internal validity due to the often-lower risk of bias [2, 7, 11, 16]. However, sometimes randomization is unfeasible because of ethical issues and technical challenges (e.g., rare diseases and medical device technologies) [9, 11, 16]. RCTs also have a limited follow-up period, misalignment with clinical practice guidelines and care pathways, as well as insufficient generalizability of findings due to their PICO limits:

• P (population): small population size, excluding eligible population, excluding population with multimorbidity, and lack of representativeness of local population (applicable to some countries)
• I (intervention): diversity of the clinical use of an intervention across different settings, such as dosing variations and sequencing interventions based on the guidelines in each jurisdiction
• C (comparators): the limited number of comparators and inappropriate comparators reflecting the routine or standard of care, inappropriate comparators in specific settings or at the time of appraisal
• O (outcome): inappropriate or unvalidated outcome(s) [9, 11, 19].

These potential limitations of RCTs cause uncertainty around safety, effectiveness, and the long-term impact of approved technologies which makes reimbursement decisions challenging. Despite pragmatic trials, single-arm trials, and indirect comparison evidence as some sources of evidence for HTA, stakeholders look to other sources of evidence like RWE. This type of evidence aims to fill the evidence gaps and inherent shortcomings of RCTs as well as minimize the uncertainty in decision-making processes [2, 5, 8, 11, 12, 14, 16, 20].

## The appeal of RWE: when, where, and how could RWE be used in HTA?

Despite the current limited use of RWE from some data sources in the HTA processes, broader integration of RWE from different sources can reduce uncertainty, thereby accelerating reimbursement decisions and patients’ access to health technologies. HTA agencies can use RWE throughout the HTA process in the initial assessment and/or reassessment of health technologies for different purposes [9, 21]. A number of specific applications have been proposed, such as: 1) understanding the safety and effectiveness of health technologies when RCT evidence is unavailable or unfeasible, 2) confirmation of RCT evidence in some situations to improve the certainty around the safety and effectiveness impact of health technologies, 3) improved understanding of the long-term impact of health technologies, 4) expanding the usage of health technologies in populations beyond those in trials. Importantly, all four of these applications can be useful in both the initial health technology assessments and the re-assessment process.

First, it is important to note that RWE from the “studies and registries” category of data sources is already used in the initial assessment to understand the safety and effectiveness of health technologies for which conducting experiments is unethical or harmful. The most common example of this type of RWE is the use of observational studies to understand the impact of surgical procedures and some medical devices as well as rare conditions. HTA bodies may employ such RWE to understand the nature and frequency of outcomes of utilizing those health technologies.

Second, HTA bodies may also use RWE from all three data categories to improve the certainty around the impact of health technologies during the initial assessment and reassessment. When the evidence from experimental studies for some technologies is not convincing enough due to uncertainty, RWE can help to fill the evidence gaps. For example, suppose the sample size is small for an experiment, as is the case for rare diseases or rare outcomes. RWE can allow HTA bodies to fill the evidence gap and make recommendations with higher levels of certainty. In turn, this information can improve certainties in decision-making processes.

Third, RWE from the post-marketing phase helps to understand the long-term (over the period of clinical trials) impact of health technologies and can be used to reassess funded technologies. Depending on the type of initial recommendation, HTA bodies can reassess their primary recommendations using RWE mainly from the “clinical records” and “unsupervised sources” categories to ensure the impact of recommended technologies on patients’ outcomes in real life for a more extended period.

Finally, RWE can also account for the expanded scope of health technologies beyond the populations involved in clinical trials. These populations, although excluded from the RCTs, can still derive benefits from these technologies. This phenomenon is observed in various health technologies upon market entry. For example, RCTs often exclude, yet in real-world conditions, many patients with chronic pain use substances.

On top of the advantages of RWE in filling RCT evidence gaps, the broader use of RWE brings other applications like confirming the Pharmacoeconomics and budget impact models and assumptions as well as improving patient engagement. Pharmacoeconomics and budget impact models and assumptions can be confirmed by employing data from all three data source categories in the assessment and reassessment process of health technologies. Likewise, incorporating RWE from “unsupervised sources” in the assessment and reassessment processes can improve understanding of patients’ values, needs and preferences after extensive use of health technologies in the market.

Depending on the source of RWE (“studies and registries,” “clinical records,” or “unsupervised sources”) the considerations to utilize RWE in the HTA processes vary. For example, using RWE from “studies and registries” focus on the scope of the study (PICO[ST](setting and time) criteria), study design, data management, methodological approach, analytical methods, and reporting are required [19]. In case the source of RWE is registries or surveys, some considerations such as data management, governance (purpose and funding source of the study and registries), quality management, and linkage need to be addressed. Using RWE from “unsupervised sources” needs to consider the scope, data security and privacy, data management, quality measurement and evaluation, and linkage. Depending on the study question, any of these data sources can be used to generate RWE on the effectiveness and safety of a technology, demographics, prevalence and incidence of a health condition, appropriateness of assumptions, patient adherence and satisfaction, patient-reported outcomes (PROs), costs, and resources used [11, 19].

Using RWE from various data sources in HTA processes for the discussed purposes in the initial assessment and reassessment of funded technologies helps decision-makers to efficiently allocate health care resources through reinvestment or disinvestment of health technologies. With the aging population, the rapidly growing number of emerging health technologies, rising costs, payers’ budget constraints, and technologies’ patent life, decision-makers need to prioritize and fund the technologies with the best value for money more than ever [22, 23]. Therefore, developing standard frameworks to use the appropriate types of RWE from each data source in the reassessment process by the HTA bodies is a crucial step for making informed decisions.

## Barriers and challenges to using RWE in HTA

The broader integration of RWE in the HTA process is a complex process and needs multistakeholder involvement. There are several barriers to using RWE from different sources in HTA processes including acceptability, transferability, reliability, validity, and generalizability of evidence generated from RWD. Concerns arise regarding the heterogeneity of the data even with standardized protocols. However, by justifying use of the data source, adhering to rigorous methodological standards, ensuring transparency, and following guidance on generating valid and high-quality RWE, confusion on disparate data can be minimized. Thus, guidance on generating valid and high-quality RWE needs to be followed when transferring RWD from one country to another. The guidance should include concise criteria for determining data quality, best practices, and validated analytical methods to address the biases when transferring RWD between countries. The other major challenges include transparency, data quality, security and privacy, and data linking. These challenges and barriers apply to many data sources but with varying levels of importance depending on the type and complexity of data sources that need to be considered [1, 2, 4, 9, 11, 14, 19, 24, 25].

Central to most of these challenges is the need for transparency that would allow for improving RWE from all categories of data sources for decision-making purposes. Greater transparency helps to improve the reproducibility and rigour of the generated RWE, enabling HTA bodies to ensure that the appropriate methods are applied to the RWE and ease the evaluation of the data and analysis quality [4, 14, 25]. Promoting transparency builds trust in the evidence-generation process and the reliability of the evidence. For example, any methods used to handle incomplete and missing data, measurement errors, incorrect data, and data misclassification are required to be clearly reported for the key variables. Such transparency can be rather complex as it may involve reporting detailed information on data sources, sharing analytical codes, and displaying full transparency on all assumptions and decisions related to the execution of the study. This level of reporting may not be common practice and involves navigating complex agreements for data access, privacy, and intellectual property [11, 25]. While transparency is essential for RWE generated from all categories of data sources, it is of even greater concern for “unsupervised sources,” which contain greater uncertainty due to the data quality of various sources.

Central to any submission using RWE, as discussed, is the quality of the RWD used. Data quality is contingent upon a combination of factors, including accuracy, reliability, robustness, and clinical relevance. The low quality and challenges of biased and incomplete data are among the main concerns in using RWE [4, 11, 12, 14, 18, 25]. These issues can apply to almost all data sources but are again more prominent with data from “unsupervised sources” because of the diversity of patients and the lack of defined standards for developers, data providers, and collectors. Accordingly, the first step in improving the quality of patients’ generated data from “unsupervised sources” is defining and implementing quality standards for each type of data source separately. This step is crucial because data insufficiency and quality would be challenging for the HTA, and even the best statistical methods cannot overcome low-quality data and, down the road, causes more uncertainty in the decision-making processes. Multistakeholder, including but not limited to HTA bodies, need to establish quality standards for each data source category and possibly each type of data source to foster an environment where producers and developers encourage the adoption of higher-quality data [18].

Data security, privacy, and access are serious issues in all patient data but are especially a major concern when leveraging the “clinical records” and “unsupervised sources” categories. Providing a secure data environment needs to follow certain IT procedures and policies, which are required to be specified and evaluated in the HTA processes. Obviously, this challenge is more applicable to digitalized data storage. The RWE from “clinical records” and, particularly, “unsupervised sources” also need to follow established regulations and privacy laws (e.g., obtaining informed consent from patients, who can be the user and how, data storage, etc.) to enable the HTA system to rely ethically on that RWE in addition to quality standards. Data sources collected with study protocols or stored at health-related organizations must also meet the minimum defined standards in each jurisdiction and not compromise the privacy of patients in the data sources.

The final major challenge in the use of RWE pertains to the need to improve and optimize data through data linkage from all categories. This often requires addressing the issue of data cleansing beforehand whenever integrating diverse data sources with heterogenous data [18, 26, 27]. HTA agencies use a range of evidence to issue their recommendations with higher levels of certainty to address payers’ needs because no one source alone can offer the required information. Sometimes, a combination of data from different sources and, occasionally, another database is required to answer the evaluation questions. For example, having drug dispensation data alone cannot shed insights on the effectiveness of a drug, but rather linking drug dispensation data with hospitalization data would allow assessing a drug’s ability to reduce hospitalizations. Thus, in the ideal world, linking data from several sources exponentially improves their collective usefulness in answering important questions. To achieve this goal, such connectivity and data linking need interoperability within health systems and even across jurisdictions, resulting in more informed decisions and better patient outcomes. System interoperability requires advanced infostructure. While many jurisdictions, like Scandinavian countries, have served as examples of data linkage and tackled these issues, it is still lacking within many other jurisdictions [28]. Such a big change needs multistakeholder involvement and standard frameworks at the health system level across all jurisdictions. The common data model can enhance the interoperability of data for evidence generation in HTA. It standardizes the structure and data format across diverse datasets, analytical tools, terminologies, etc., which fosters transparency and confidence [29].

## Conclusion

RWE can be synthesized from different categories of data sources, which have been limitedly used in the HTA processes so far, but broader integration of RWE in the HTA processes to improve certainty in reimbursement decisions is needed. Apparently, the broader use of RWE needs to overcome several challenges and barriers. To tackle significant barriers to using RWE from different sources in HTA, agencies need to establish reporting standards, define quality standards, and champion the development of methodologies and standard frameworks, perhaps specific to each data source category. RWE has the potential to fill some evidence gaps and reduce the uncertainty around the costs as well as the long-term and uncontrolled impact of health technologies in real-world conditions. The reassessment of reimbursed health technologies using RWE by HTA agencies further supports decision-makers in efficiently allocating scarce health resources and making more informed reimbursement decisions. After the reassessment of health technologies by HTA agencies, only the technologies that have truly improved the health outcomes in the relevant population and have a good value for money in the real world may be publicly funded by the decision-makers. Health systems, depending on their current health policies and structures, may need to define new reimbursement strategies or adapt their current funding policies for reinvestment or disinvestment when RWE from all categories is routinely used on a broader scale in the HTA processes.
